# Facilitated recruitment of mesenchymal stromal cells by bone marrow concentrate and platelet rich plasma

**DOI:** 10.1371/journal.pone.0194567

**Published:** 2018-03-22

**Authors:** Hannah L. Holmes, Brooke Wilson, Julian P. Goerger, Jesse L. Silverberg, Itai Cohen, Warren R. Zipfel, Lisa A. Fortier

**Affiliations:** 1 Department of Clinical Sciences, Cornell University, Ithaca, NY, United States of America; 2 Department of Biomedical Engineering, Cornell University, Ithaca, NY, United States of America; 3 Department of Physics, Cornell University, Ithaca, NY, United States of America; Chung-Ang University College of Engineering, REPUBLIC OF KOREA

## Abstract

**Background:**

Biologics containing growth factors are frequently used to enhance healing after musculoskeletal injuries. One mechanism of action is thought to be though the ability of biologics to induce homing and migration of endogenous mesenchymal stromal cells (MSCs) to a target tissue. However, the ability of biologics to stimulate chemotaxis (directed migration of cells) and chemokinesis (increase rate of cell migration) of MSCs is unknown.

**Hypothesis/Purpose:**

The aim of this study was to directly compare the ability of biologics including platelet rich plasma (PRP) and bone marrow concentrate (BMC) to induce MSC migration. The hypothesis was that leukocyte-low platelet rich plasma (L^lo^ PRP) would induce migration to a greater extent than leukocyte-high platelet rich plasma (L^hi^ PRP) or BMC.

**Methods:**

Bone marrow-derived MSCs were isolated from 8 horses. Migration of MSCs toward a biologic (BMC, L^lo^ PRP, and L^hi^ PRP) or the positive control platelet derived growth factor (PDGF) was continuously traced and measured for 24hrs using time-lapse microscopy and a microfluidics device. Cell migration, chemotaxis and chemokinesis were determined by measurements of displacement, number of cells migrated, and cell flux.

**Results:**

All biologics resulted in a significantly greater percentage of MSCs migrated compared to the positive control (PDGF). MSCs migrated further toward BMC compared to L^lo^ PRP. Cell migration, measured as cell flux, was greater toward BMC and L^hi^ PRP than L^lo^ PRP.

**Conclusion:**

The biologics BMC and L^hi^ PRP elicit greater chemotaxis and chemokinesis of MSCs than L^lo^ PRP. However, all biologics recruited the same number of MSCs suggesting that differences in other regenerative effects, such as growth factor concentration, between biologics should be strongly considered when choosing a biologic for treatment of musculoskeletal injuries. The results of this study have the potential to reduce the need, risks, and costs associated with MSC culture and delivery.

## Introduction

Mesenchymal stromal cell (MSCs) implantation can improve tissue repair and patient function after musculoskeletal injury.[1–7] However, autologous MSC therapy is costly and time-consuming, requiring several weeks of culture to acquire sufficient cells for administration. This time requirement for culture also delays patient treatment.[8] Use of allogeneic cells might circumvent these issues, but concerns remain about their antigenicity.[9–11] Further limiting the implementation of MSC therapy in patients is the lack of approval for use in humans by many governing regulatory agencies throughout the world. An alternative means to provide MSC therapy for patients is the use of regenerative medicine approaches to recruit endogenous tissue MSCs that are juxtaposed to the site of injury through the application of biologics.[1,2,12]

Biologics such as platelet rich plasma (PRP) and bone marrow aspirate concentrate (BMC) have been used to enhance healing of musculoskeletal injuries.[13–15] In the area of osteoarthritis (OA), there are several level 1 studies demonstrating the pain relieving, symptom modifying, and chondroprotective effects of PRP following direct injection into arthritic knees.[16–18] Bone marrow concentrate started as a method for repair of cartilage defects,[19] but more recently is used in a similar manner as PRP for direct injection into a knee affected with OA[20–22] with less evidence than PRP, yet good evidence to support its use. Both of these biologics contain bioactive growth factors such as transforming growth factor β-1 (TGFβ-1), TGFβ-3, and platelet-derived growth factor (PDGF), which are thought to be in part responsible for the healing effects of biologics through their characteristic ability to promote healing by stimulating cell migration, cell proliferation, angiogenesis, and matrix synthesis.[23,24] There are clear differences and relative advantages/disadvantages to the use of PRP or BMC with respect to bioactive molecules, and that BMC, but not PRP contains MSCs.[25,26] This has led some to consider BMC as superior to PRP because it contains stem cells. However, obtaining BMC necessitates a moderately invasive bone marrow aspirate (BMA) while PRP on requires a simple blood sample. In addition to the relative ease of generating PRP, one study demonstrated that PRP can stimulate chemotactic migration of MSCs across a transwell membrane,[27] which might suggest that the presence of MSCs in BMC is not a significant advantage over PRP if PRP can recruit MSCs. The purposed of this study was to directly compare and quantify the ability of biologics (PRP, BMA, and BMC) to induce MSC migration.

In this study, a microfluidics device and time-lapse microscopy were used to measure and compare the differing ability of biologics to induce chemotaxis or chemokinesis of MSCs. The goal of this study was to determine which biologic induced the greatest migration of MSCs and would therefore be an optimal candidate for use in *in vivo* regenerative medicine. The biologics used in this study included BMA, BMC and two types of PRP: leukocyte high platelet rich plasma (L^hi^ PRP; leukocyte concentration in PRP is greater than starting blood sample) and leukocyte low platelet rich plasma (L^lo^ PRP; leukocyte concentration in PRP is less than starting blood sample). Two types of PRP were investigated because neutrophils can be detrimental to tissue repair,[28,29] and L^lo^ PRP is thought to result in improved matrix homeostasis and tissue repair compared to L^hi^ PRP.[30,31] The hypothesis for this study was that all the biologics would stimulate chemotactic migration of MSCs, but L^lo^ PRP would be the optimal chemotactic biologic due to the low concentration of leukocytes.

## Materials and methods

All animal use was approved by the Cornell University Institutional Animal Care and Use Committee. Intravenous sedation and local anesthetic deposited in the skin and down to the sternum was used for bone marrow aspirate procedures. For blood draws, sedation was only used when necessary according to the temperament of the animal.

### Mesenchymal stem cell isolation

Bone marrow aspirate was obtained from the sternum of eight mature horses (ages 2–19 years) into syringes containing heparin to a final concentration of 100 units heparin/ml bone marrow aspirate. Neither the aspirates nor resultant cells were ever pooled. Aspirate were processed using Ficoll (GE Healthcare, Piscataway, NJ) density gradient centrifugation. Cells were expanded on tissue culture plates using complete stem cell medium (Dulbucco’s Modified Essential Medium, 10% FBS, penicillin and streptomycin, hepes buffer, L-glutamine, bFGF) at 37°C in a humid, 5% CO_2_/air incubator. Cells were washed with PBS and medium was changed every third day until they reached 80–90% confluence. Cells were then lifted with Accumax (EMD Millipore Corporation, Billerica, MA) and used at first passage. Cells were confirmed to undergo tri-lineage differentiation using the method previously reported by our laboratory.[32]

#### Bone marrow aspirate and bone marrow aspirate concentrate

Bone marrow aspirate was obtained as described above and processed using SmartPReP 2 Technology (Harvest Technology Corp, Plymouth, MA) to generate BMC. Samples of BMA and BMC were aliquoted into 1ml cryovials and frozen at -80°C. Prior to freezing, complete blood counts for BMA and BMC were performed in an accredited clinical pathology laboratory.

### Platelet rich plasma

Two commercial systems were used to generate PRP. Blood was drawn into a syringe to a final concentration of 1% acid citrate dextrose. The Double Syringe Autologous Conditioned Plasma System (Arthrex Inc, Naples, FL) was used to generate L^lo^ PRP. The GPS III Platelet Separation system (Biomet Inc, Warsaw, IN) was used to generate L^hi^ PRP. Both types of PRP were aliquoted into 1ml cryovials and frozen at -80°C. Samples of blood and PRP were retained for complete blood counts and differentials which were performed in an accredited clinical pathology laboratory.

### Controls

Human recombinant platelet derived growth factor AB (PDGF-AB) (Life Technologies, Grand Island, NY) was used as the positive control to discriminate between poorly migrating cells and ineffective biologics.[33] Pilot studies were performed to test the chemoattractant ability of several agents at various concentrations, including PDGF-AB, TGFβ-3, and serum. Complete stem cell media with 10% fetal bovine serum (FBS) was used as the neutral control (NC). Results indicated that PDGF-AB induced the greatest MSC migration, consistent with previous reports.[24,34]

### Microfluidics device preparation and measurements of cell migration

**Fig 1:**
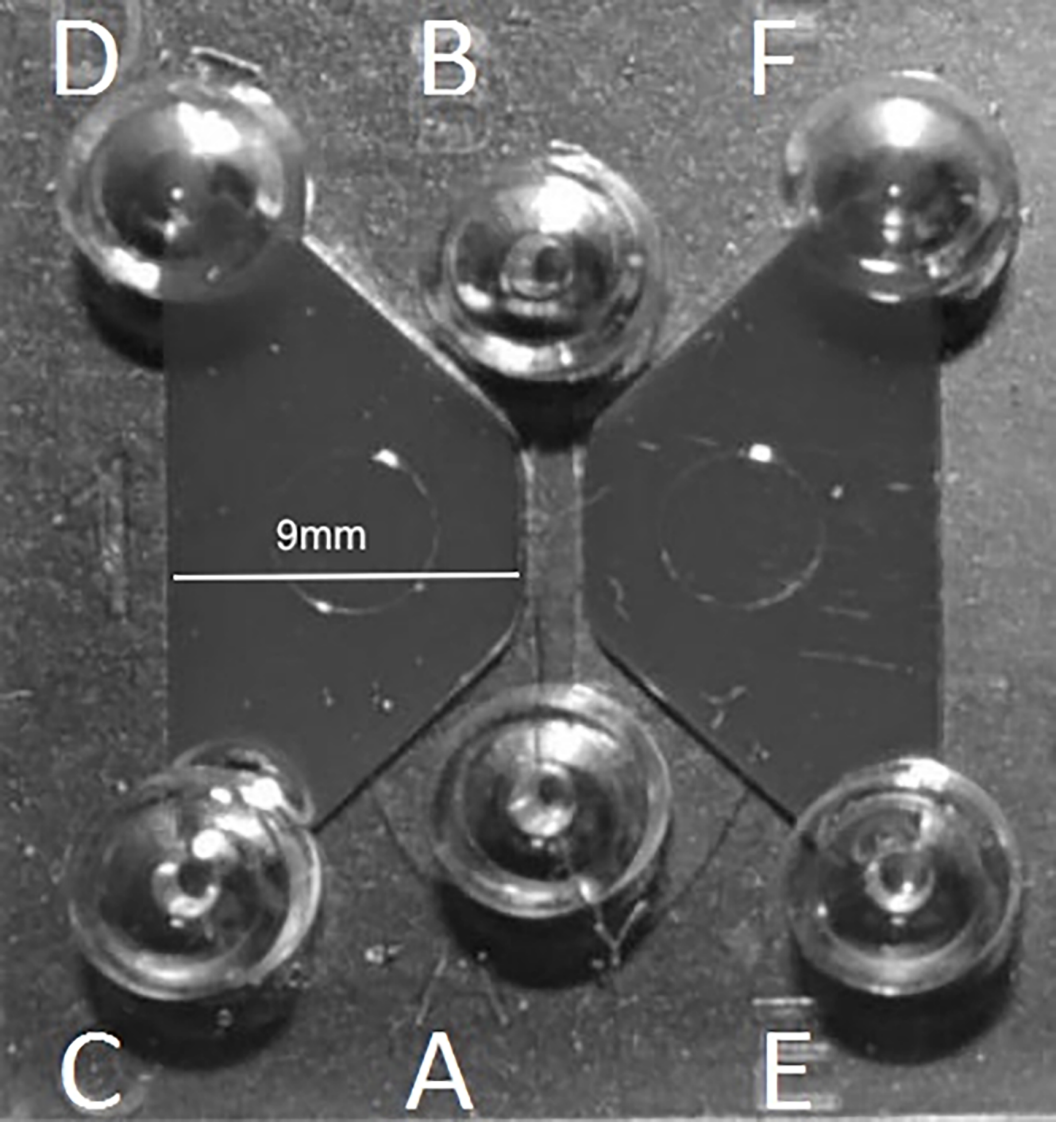
The μ-slide chemotaxis device. The area between ports A and B is the observation area where MSCs are seeded. The trapezoid shaped area between ports A, B, C, and D is the reservoir well where putative chemoattractants are placed. The trapezoid shaped area between ports A, B, E, and F is the reservoir well where the neutral control (stem cell media with 10% fetal bovine serum) is placed.

To prevent air bubble formation in the microfluidics device (μ-slide chemotaxis devices, Ibidi LLC, Verona, WI), complete stem cell media, and stem cell media with 1% FBS were incubated for 24 hours prior to use to allow for CO_2_ equilibration. Passage 1 MSCs were washed with PBS, centrifuged and re-suspended at 27*10^3 cells/cm^3^ in 10% FBS media. The observation area was filled with 6μl of cell suspension (**Fig 1**). The device was placed in a petri dish and incubated for 2–3 hours to allow MSCs adherence. Biologics were thawed and centrifuged at 12,000*g* for 20 minutes to pellet cell debris. Biologic supernatant and PDGF-AB were incubated with the device to allow CO_2_ content to equilibrate.

Reservoir wells of each device were then filled with 60μl of 1% FBS media then 10μl of biologic was pipetted into port D while 5μl of 1% FBS media was aspirated from port C to draw the biologic into the reservoir well (**Fig 1**). Similarly, 10μl of 10% FBS NC media was placed in port F while 5μl of 1% FBS media was aspirated from port E so that a direct competition of chemoattraction could be measured between a biologic and the NC.

The device was imaged within the CO_2_ incubator on a lab-built miniaturized bright field microscope with red light illumination provided by an LED and image acquisition by a 4X objective (Olympus America, Center Valley, PA) and USB 1.3 MP CCD camera (Point Grey, Richmond BC). An automated XY translation stage was used to move between observation regions in six different wells so that all treatments were imaged per experiment, and eight 24 hour imaging experiments were performed. Control software was written in Visual Basic under Microsoft Visual Studio 8 (Microsoft Corp, Redmond, WA). Images were acquired every five minutes for a period of twenty four hours. Time-lapse images were analyzed by manually tracking migratory patterns of individual cells by use of a custom code written in Image J (NIH, Bethesda, MD). Cells were tracked up to the point of division, death, migration out of the center well, or 24 hours of migration.

A custom code written in Matlab (MathWorks Inc, Natick, MA) was used to determine displacement of each cell from its location at time 0 to their final location.

Cell coordinate data were separated into two groups: those that migrated toward the biologic and those that migrated toward the NC. The average displacement of individual cells in each group was calculated to provide overall displacement for each experiment. The number of cells migrating in each direction was also recorded. Cell flux was calculated by the following formula:

$$Cell\;Flux=\frac{\%\;\mathrm{of}\;\mathrm{cells}\;\mathrm{migrated}\;\mathrm{towards}\;a\;\mathrm{treatment}*\mathrm{average}\;\mathrm{distance}\;\mathrm{migrated}}{24\;\mathrm{hours}}$$

with units of (%μm/hr). This calculation was used as a metric to quantify movement of MSCs within the device. The experiment was repeated for each of the 8 animals.

**Fig 2:**
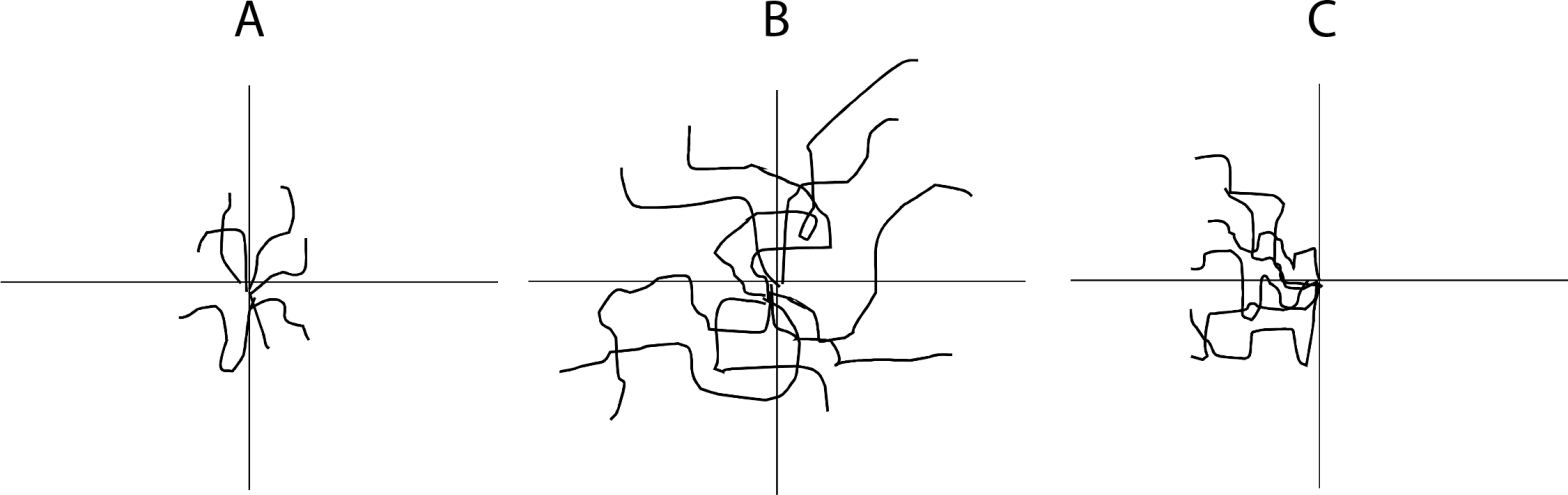
Chemotaxis compared to chemokinesis. Black lines indicate cell trajectories. A) Cells are not stimulated by any factors, they move randomly. B) Cells are stimulated by a chemokinetic factor. They have increased the rate of migration, but do not move in a particular direction. C) Cells are stimulated by a chemotactic factor. Their rate of migration has not increased, but directed migration is occurring either up or down a chemical gradient.

The percentage of cells migrated was used as a measure of the ability of biologics to act as chemotaxis agents for MSCs. Chemotaxis refers to the ability to cause a directed migration of cells.[35] Cells influenced by a chemotactic factor will move along a chemical gradient (**Fig 2**). Displacement of MSCs was used to measure the ability of a biologic to stimulate chemokinesis. Chemokinesis refers to the ability to cause an increase in rate of migration.[30] Cells influenced by a chemokinetic factor will move further in the same amount of time as cells that were not influenced by the chemokinetic factor, but not in any specific direction.

### Statistical analysis

Outcome variables included number of cells migrated, cell displacement, and cell flux. The number of cells migrated was normalized by calculating percentage migrated in order to control for differences in seeding density of MSCs between experiments. Each experiment allowed for direct competition of a putative chemoattractant against the NC. Each experimental device had a NC, resulting in five NC groups existed. Outcome variables from each NC experiment were compared and were not significantly different from each other, so the NC data was averaged for further statistical analyses. The NCs were compared using a Kruskal-Wallis one-way ANOVA by ranks using JMP Pro 11 (SAS Institute Inc, Cary, NC). Independent variables were NC, PDGF-AB, BMA, BMC, L^lo^ PRP and L^hi^ PRP. Dependent variables, including percent migrated, displacement, and flux were analyzed using a Kruskal-Wallis one-way ANOVA by ranks. A p-value of < 0.05 was considered significant. Post hoc comparisons between groups were made using the Wilcoxon rank-sum test with a downward adjustment in the p-value to compensate for the increased chance of type-I error with multiple comparisons. A p-value of < 0.01 was considered significant.

## Results

### Percentage of cells migrated

**Fig 3:**
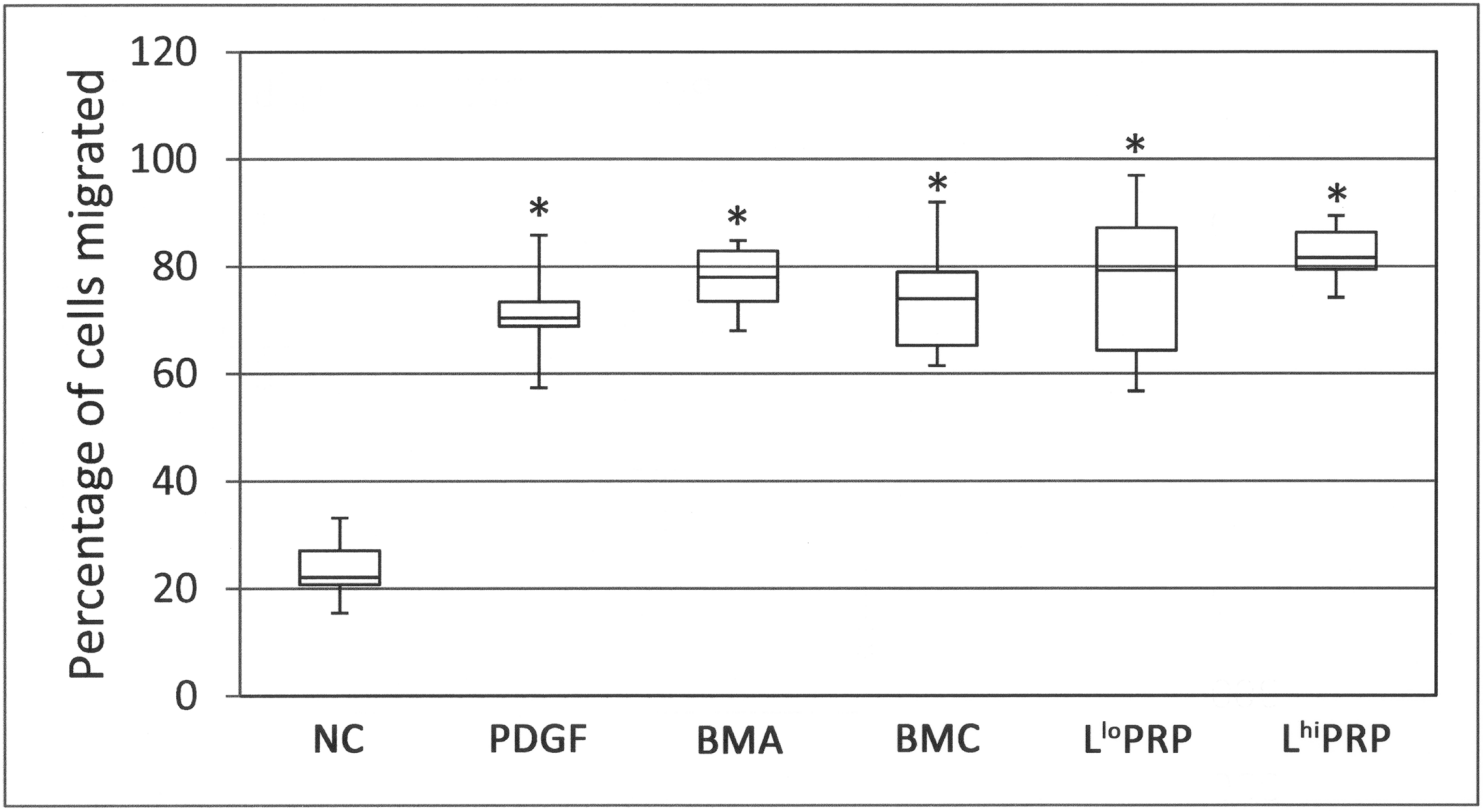
Percentage of cells migrated toward a biologic. All putative chemoattractants resulted in significantly greater percentage of cells migrated (asterisks) than the NC. Data are represented as median with maximum and minimum values; n = 8. NC = neutral control, PDGF = platelet derived growth factor, BMA = bone marrow aspirate, BMC = bone marrow aspirate concentrate, L^lo^ PRP = leukocyte low platelet rich plasma, L^hi^ PRP = leukocyte high platelet rich plasma. Significance was determined by a Kruskal-Wallis followed by Wilcoxon multiple comparison post-hoc test. A p-value < 0.01 was considered significant.

**Fig 4:**
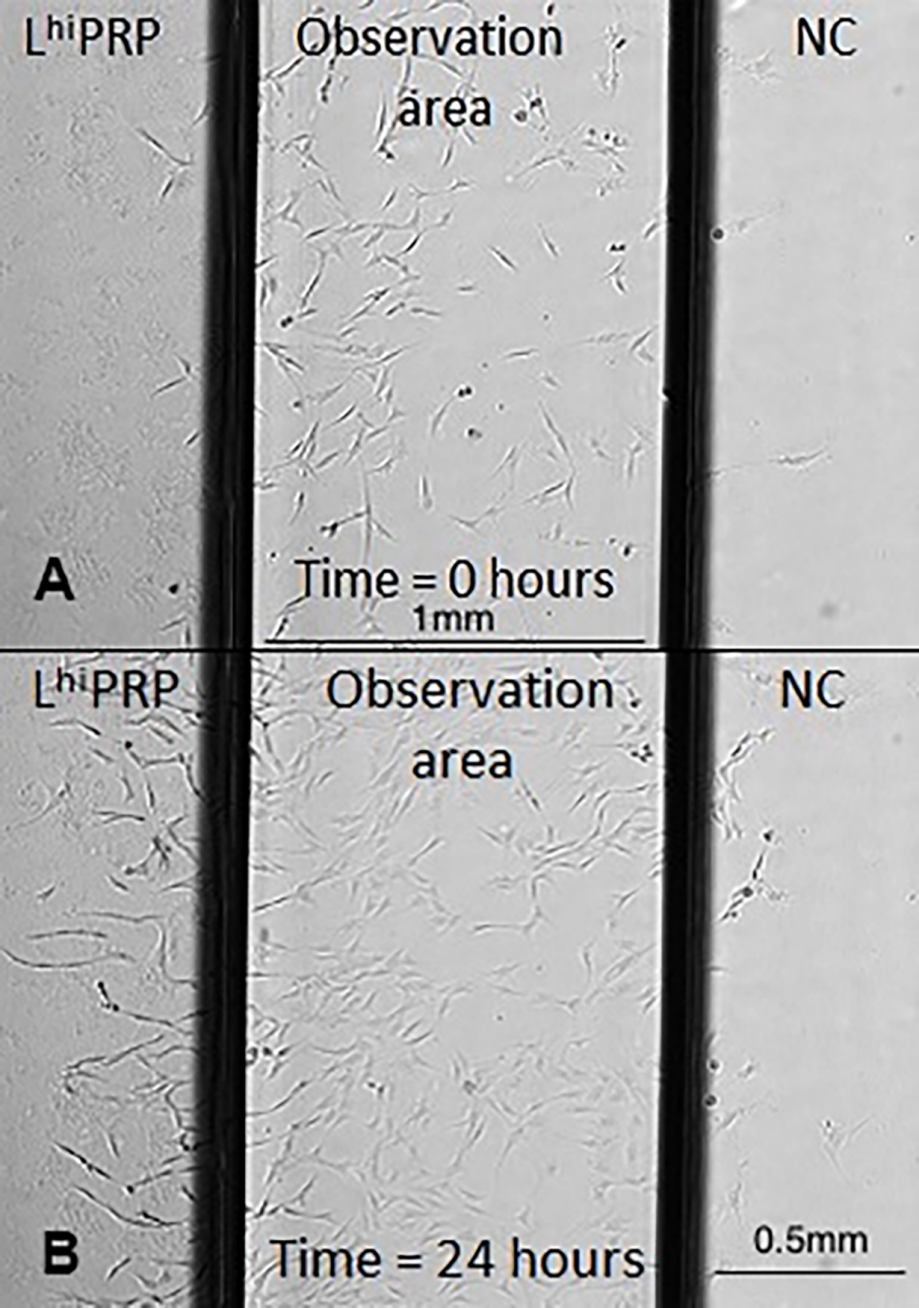
Cell migration images within the microfluidics μ- slide chemotaxis device at (A) time = 0hrs, and (B) time = 24hrs. Cells show preferential migration toward leukocyte high platelet rich plasma (L^hi^ PRP) compared to the neutral control (NC).

Biologics attracted 3–4 times the percentage of cells compared to the NC (**Fig 3**; p < 0.001). The positive control, PDGF, also attracted significantly more cells compared to the NC (p < 0.001). None of the biologics were different from each other. Visual inspection of individual frames from time-lapse videos showed cells migrating toward biologics in preference to the NC (**Fig 4**). Video images demonstrate migration of MSCs toward the biologics (**S1 Video**).

### Cell displacement

**Fig 5:**
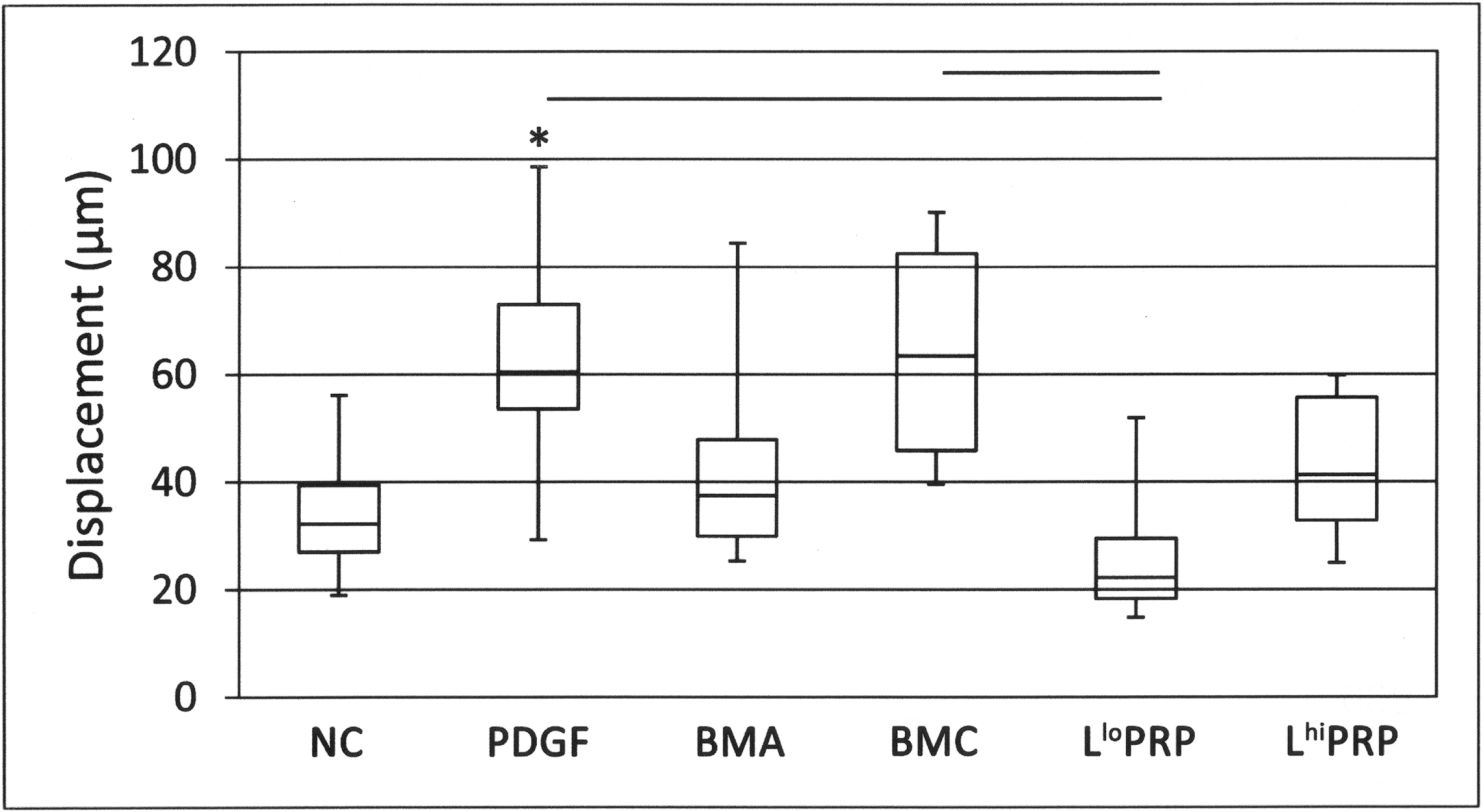
Displacement of cells toward a putative chemoattractant. BMC and PDGF resulted in greater displacement in comparison to L^lo^ PRP (horizontal bars). PDGF resulted in greater displacement compared to the NC (asterisk). Data are represented as median with maximum and minimum values; n = 8. NC = neutral control, PDGF = platelet derived growth factor, BMA = bone marrow aspirate, BMC = bone marrow concentrate, L^lo^ PRP = leukocyte low platelet rich plasma, L^hi^ PRP = leukocyte high platelet rich plasma. Significance was determined by a Kruskal-Wallis followed by Wilcoxon multiple comparison post-hoc test. A p-value < 0.01 was considered significant.

Bone marrow concentrate stimulated 2.5 times more displacement than L^lo^ PRP (**Fig 5**; p = 0.005). Similarly, PDGF stimulated 2.4 times more displacement than L^lo^ PRP (p = 0.002). Displacement of cells was 1.9 times greater toward PDGF than toward the NC (p = 0.01).

### Cell flux

**Fig 6:**
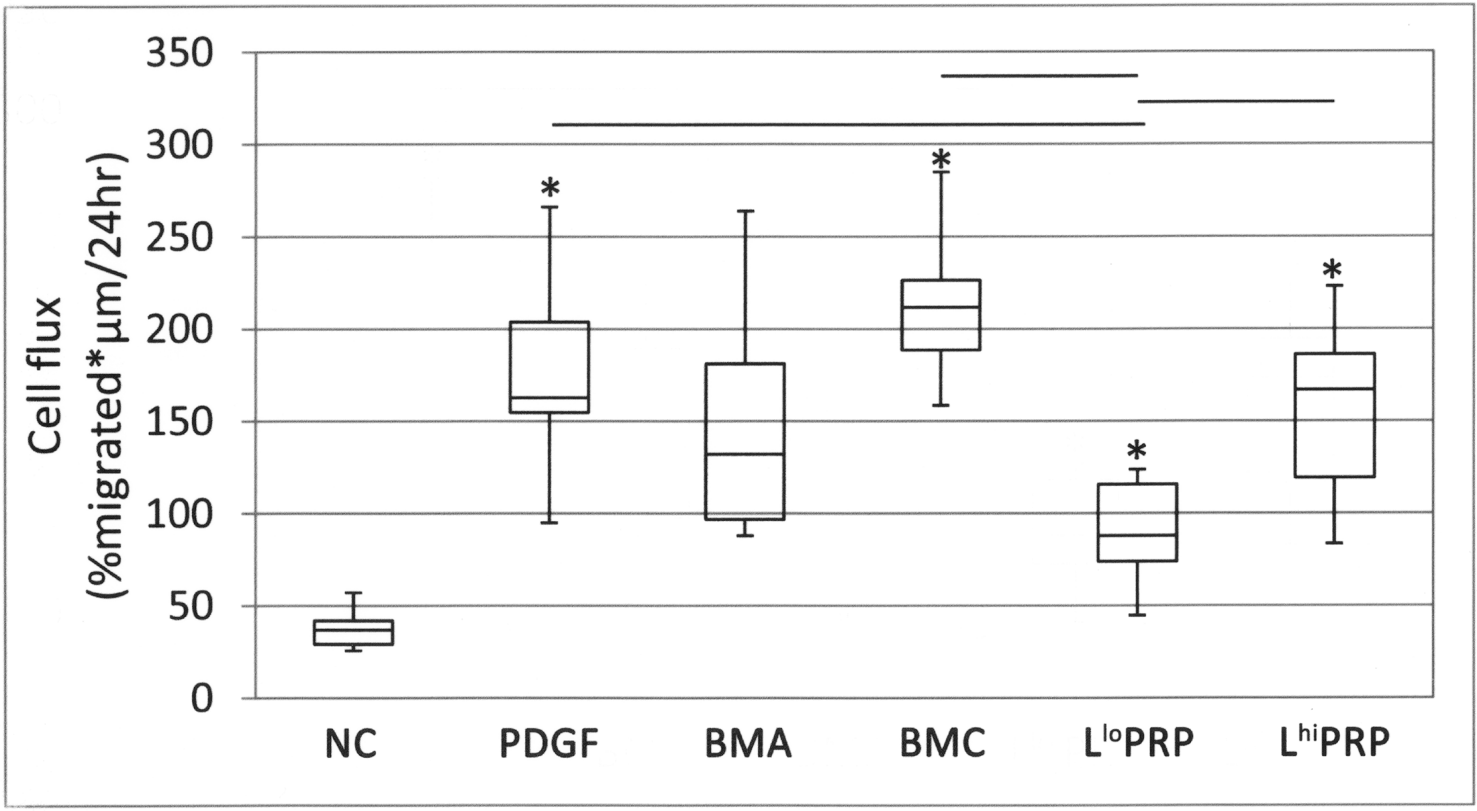
Cell flux toward a putative chemoattractant. PDGF, BMC, L^hi^ PRP resulted in significantly more cell flux than L^lo^ PRP (bars). PDGF, BMC, L^lo^ PRP, L^hi^ PRP resulted in significantly greater cell flux compared to the NC (asterisks). Data are represented as median with maximum and minimum values; n = 8. NC = neutral control, PDGF = platelet derived growth factor, BMA = bone marrow aspirate, BMC = bone marrow aspirate concentrate, L^lo^ PRP = leukocyte low platelet rich plasma, L^hi^ PRP = leukocyte high platelet rich plasma. Significance was determined by a Kruskal-Wallis followed by Wilcoxon multiple comparison post-hoc test. A p-value < 0.01 was considered significant.

Cell flux was 2.4 times greater toward BMC than L^lo^ PRP (**Fig 6**; p = 0.002). Cell flux toward L^hi^ PRP was 1.8 times greater than toward L^lo^ PRP (p = 0.01). BMC induced 5.5 times greater cell flux of the NC (p = 0.0009). L^hi^ PRP stimulated 4.1 times more cell flux than the NC (p = 0.001) and L^lo^ PRP stimulated 2.2 times more cell flux than the NC (p = 0.01). Cell flux toward PDGF-AB was 5.2 times greater than cell flux toward the NC (p < 0.001). PDGF-AB resulted in 2.3 times more cell flux than L^lo^ PRP (p = 0.002).

## Discussion

Contrary to our hypothesis, BMC and L^hi^ PRP resulted in enhanced MSC migration compared to L^lo^ PRP. Chemokinesis, measured as cell displacement, was greatest toward BMC, while L^hi^ PRP resulted in slightly more chemotaxis which was measured as percentage of migrated cells. Because flux incorporates both of these values, there was no significant difference between BMC and L^hi^ PRP. Although L^lo^ PRP did not result in as much cell flux or displacement as the other biologics, it has an equivalent ability to induce chemotaxis of MSCs.

Both chemotaxis and chemokinesis factors are important in determining the optimal biologic for enhanced stem cell recruitment. The optimal biologic would induce directed migration of cells towards the wound and increase the speed at which cells could reach the wound. Growth factors range in their ability to stimulate chemotaxis and chemokinesis. For example, C-X-C motif chemokine 12 (CXCL12) can promote chemotaxis but not chemokinesis in human blood cord stem cells.[36] Insulin-like growth factor (IGF) I and II can promote chemotaxis and chemokinesis of malignant mesothelioma cells.[37] Platelet derived growth factor is a known chemotactic growth factor for cells of mesenchymal origin.[33] Growth factor interactions can also alter the response of a cell. For example, PDGF-AB results in increased expression of IGF-I receptors.[38] All biologics used in this study would contain comparable concentrations of IGF-I, because of the basal concentration of IGF-I in blood and bone marrow.[39] Differences in concentrations of PDGF and other growth factors in biologics was likely the cause of the varying responses seen by MSCs exposed to biologics. The percentage of cells migrated toward L^lo^ PRP was not different from any of the other biologics. However, it induced the least amount of cell displacement. In other words, the chemotactic ability of L^lo^ PRP was equivalent to the other biologics studied, but it was less able to induce chemokinesis compared to L^hi^ PRP, BMA, or BMC. Cell flux is a particularly useful measure of migration because it represents both chemotactic and chemokinetic capability of biologics. BMC and L^hi^ PRP stimulated more cell flux than L^lo^ PRP.

A limitation of this study was that the effect of biologics on cell division was not measured. Growth factors not only cause cell movement, but also act as mitogens. The mitogenic capability of a biologic could have an effect on cell flux. When a cell divides, it produces two daughter cells. If both cells migrate toward the chemoattractant, then the percentage of cells migrated would change and cell flux which would increase for that direction. Future studies, which look at MSCs over the time scale of multiple divisions, would be interesting as they would allow for observation of the long-term response of MSCs to biologics. In the experiments of this study, cells were tracked for twenty-four hours because MSCs became too confluent to individually distinguish. When cells were plated at a lower concentration to plan for longer observation periods, they did not migrate until they became more confluent. This suggests that cell-cell interaction is necessary to promote migration. Interesting phenotypes were observed in early experiments that were allowed to run for longer periods of time. Cells exposed to BMA or BMC tended to roll into long sheets of cells and then form spheroids. Some studies have deliberately induced spheroid formation[40] which can occur when cells become confluent or are nutrient deprived. Formation of spheroids allows stem cells to maintain viability in serum-free or hypoxic conditions. This is consistent with the present study when cells reached confluence and were imaged for three or more days.

Biologics are a complex mixture of numerous bioactive molecules thought to be important for functional tissue regeneration. Because PRP and BMC have different anabolic and catabolic molecular compositions,[26] it is not uncommon for them to be investigated in the laboratory or clinic as a combination product.[41–43] It would be interesting to investigate the chemotactic properties resulting from the combination of PRP and BMC to understand if there is added value with respect to attraction of stem cells if PRP and BMC are delivered together as opposed to single product. Because all biologics tested in this study resulted in chemotaxis and/or chemokinesis of MSCs with some subtle differences, there is a suggestion that combining the two biologist might differently affect MSC migration. In vivo studies are needed to determine the effects that biologics have on the recruitment of MSCs within an injured tissue environment, and how this functionally affects tissue repair and patient outcome. MSC tracking studies using fluorescent labels, Qtracker beads, positron emission tomography–computed tomography (PET-CT) and numerous other methods[44–48] are available with many more in development, and could be used to determine which biologic or combination of biologic results in the greatest stem cell migration in vivo. These in vivo MSC migration studies would further our understanding of the mechanism of action for the biologics in tissue repair which should further optimize the use of biologics in the field of regenerative medicine.

## Supporting information

S1 Video Migration of mesenchymal stem cells A) BMA is on the left, NC is on the right. B) BMAC is on the left, NC is on the right. C) L^lo^PRP is on the left, NC is on the right. D) L^hi^ PRP is on the left, NC is on the right.
(WMV)
